# Visualizing mechanistic models by integrating site-specific molecular details into reaction networks

**DOI:** 10.3389/fmolb.2025.1681081

**Published:** 2025-11-20

**Authors:** Dan Vasilescu, James C. Schaff, Ion I. Moraru, Michael L. Blinov

**Affiliations:** Center for Cell Analysis and Modeling, University of Connecticut School of Medicine, Farmington, CT, United States

**Keywords:** systems biology, mathematical modeling, rule-based modeling, reaction network, visualization, SBGN, Virtual Cell, VCell

## Abstract

Mechanistic modeling in biology aims to describe biological processes based on details on molecular mechanisms and interactions. Rule-based mechanistic modeling enables the simulation of biological systems while explicitly accounting for molecular details, such as protein domains and their specific interactions. Traditionally, mechanistic models are visually represented by reaction or pathway diagrams that depict transformations and modifications of chemical species. In contrast, rule-based descriptions are effective to encode the detailed specificity of individual interactions (e.g., how phosphorylation at a particular residue affects binding affinity elsewhere in the same protein complex) in a compact and precise form, but are complicated to integrate into comprehensive visual representations. Here, we introduce **Molecular Process Diagrams**, an approach to embed rule-based specificity directly within reaction network diagrams. Our method highlights three fundamental elements: interacting molecular complexes, molecular sites directly modified by a rule, and molecular sites that modulate but are not directly modified by interactions (e.g., phosphorylation-dependent affinity changes). Implemented at multiple resolution levels within the Virtual Cell (VCell) software, these diagrams maintain pathway-like clarity while accurately reflecting detailed molecular interactions. Additionally, we demonstrate compatibility with Systems Biology Graphical Notation (SBGN) process diagrams, ensuring standardized visual conventions.

## 1 Introduction

Mechanistic kinetic models are critical for predicting dynamics and understanding mechanisms underlying various biological processes, as biomolecular interactions govern most regulatory mechanisms. Large biomolecules such as proteins, RNA, and DNA typically contain multiple functional components, including phosphorylation sites and domains like SH2/PTB (Pawson and Nash, 2003; Pawson, 2004; Seet et al., 2006; Olsen et al., 2006). Importantly, interactions among biomolecules are often dependent on site-specific molecular details. For example, early studies on receptor tyrosine kinase signaling, such as the Epidermal Growth Factor (EGF) receptor pathway, demonstrated that binding of adapter proteins depends on phosphorylation of specific receptor tyrosine residues and the corresponding affinity of adapter protein domains (SH2 or PTB) (Kholodenko et al., 1999). Similarly, IgE receptor (FcεRI) signaling in allergic responses depends on site-specific phosphorylation events required for kinase Syk activation, which is conditioned by phosphorylation of tyrosine residues in the linker and activation loop regions by other kinases (Siraganian et al., 2002; Chylek et al., 2014).

As experimental information about site-specific interactions continues to accumulate, models incorporating such details have become increasingly relevant. However, integrating site-specific details into conventional mechanistic models—which explicitly represent reaction networks—often results in combinatorial explosion, generating excessively large sets of molecular species and reactions (Hlavacek et al., 2006; Mayer et al., 2009). For instance, the EGF receptor alone has nine independently phosphorylated tyrosines, leading to 2^9^ = 512 distinct receptor states. Enumerating each reaction explicitly to construct deterministic or stochastic mathematical models quickly becomes impractical, highlighting an intrinsic limitation of conventional modeling frameworks.

Rule-based modeling methods address this combinatorial complexity by providing compact, precise, and scalable representations of biomolecular interactions (Hlavacek et al., 2006; Blinov et al., 2004; Sneddon et al., 2011; Chylek et al., 2015; Harris et al., 2016). Multiple rule-based models are published every year. Just in 2025, there are at least three rule-based models: by Millan et al. (2025), Bartol et al. (2025), and Wan et al. (2005). Such models specify rules governing interactions among multi-site molecules, capturing all necessary site-specific details succinctly (Chylek et al., 2014). Nevertheless, the detailed complexity captured by rule-based models must be communicated clearly to the human user for effective interpretation, evaluation, and reuse. Conventional reaction network diagrams, which are commonly used to illustrate biological pathways, generally can be used to visualize only reaction networks explicitly generated from rule-based models. As a result, they are limited to visualizing relatively small models. In contrast, rule-based models can generate networks containing hundreds to thousands of chemical species, and in some cases may even yield potentially infinite networks, such as those arising from polymerization. Furthermore, conventional reaction networks are not designed to represent the internal constituents of chemical species. By comparison, networks generated through rule-based approaches capture an important feature: each species is defined by its molecular composition, represented as a connected set of molecules, where individual molecules may contain additional modification sites. Reaction rules then specify changes in connectivity and/or modification states at these sites. Omitting these important features—for example, the ability to track an adapter protein as it progresses through a set of rules—limits the insights that reaction networks can provide, even in cases where they are applicable for visualizing networks generated by rule-based models.

To address this visualization challenge, we started from the rule-based modeling capabilities previously integrated into the widely used Virtual Cell (VCell) modeling and simulation platform (Schaff et al., 1997; Moraru et al., 2008). Although the VCell implementation included a graphical user interface for visually specifying reaction rules (Schaff et al., 2016; Blinov et al., 2017), it shared a fundamental limitation common to existing rule-based modeling tools: rules could not be collectively represented in a diagram resembling traditional reaction networks or pathways.

In this manuscript, we introduce **Molecular Process Diagrams (MPDs)**, a visualization approach designed specifically to bridge the gap between detailed rule-based specifications and conventional pathway diagrams. MPDs integrate rule-based modeling details, including molecular site-specific interactions, into familiar reaction network visualizations, thus maintaining intuitive clarity without sacrificing precision.

In the following sections, we provide a brief background on rule-based modeling and review existing visualization approaches. We then describe the core principles of MPDs and illustrate their implementation within Virtual Cell. Finally, we discuss how MPDs align with established visualization standards in the community—particularly Systems Biology Graphical Notation (SBGN) (Le Novère et al., 2009)—and propose compatible conventions within the SBGN Process Diagram format.

## 2 Methods

### 2.1 Reaction networks

**FIGURE 1:**
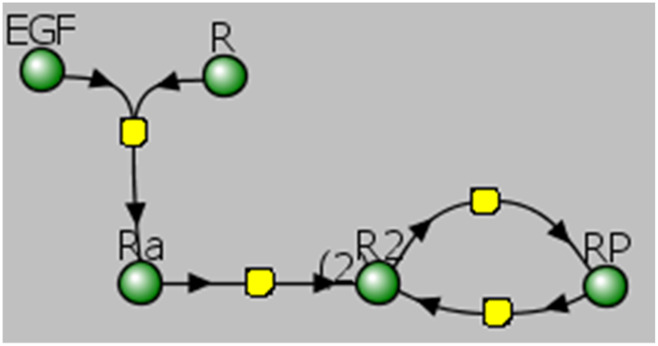
Visual representation of a reaction network created in Virtual Cell (VCell). Ligand EGF binds receptor R (EGFR) forming the complex Ra (EGF-bound EGFR), which subsequently dimerizes to produce R2 (dimeric EGFR complex). This dimer then undergoes phosphorylation, resulting in the fully phosphorylated receptor dimer, RP.

Traditionally, biological pathways are depicted as bipartite graphs, where nodes represent either molecular entities or processes, connected by edges indicating participation. Typically, the mathematical representation of a pathway mirrors its visual depiction. For example, the initial signaling events involving the EGF receptor (EGFR) described in multiple modeling papers such as Kholodenko et al., 1999; Schoeberl et al., 2002; Blinov et al., 2006a; Huang et al., 2017, include the following steps: (1) ligand (EGF) binding to EGFR; (2) dimerization of receptor-ligand complexes; (3) receptor tyrosine transphosphorylation; and (4) dephosphorylation of unprotected receptor sites by phosphatases. Figure 1 shows a reaction network corresponding to these events, created within the VCell modeling environment. In conventional modeling software tools such as VCell (Schaff et al., 1997), COPASI (Hoops et al., 2006), or CellDesigner (Funahashi et al., 2003), each chemical species and reaction must be explicitly and manually specified and localized to appropriate compartments (membranes or volumes). However, manual specification inherently limits the complexity and scale of representable models due to the rapidly growing number of potential species and interactions.

### 2.2 Rule-based approach

**FIGURE 2:**
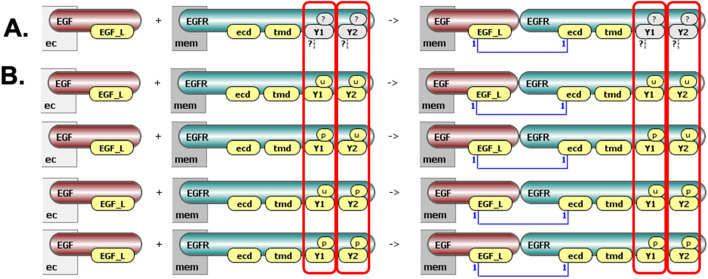
Visualization of a ligand-receptor binding rule in VCell. **(A)** This rule has two reactant patterns (ligand EGF and receptor EGFR) and one product pattern (EGF-EGFR complex). EGFR contains four interaction sites: extracellular domain (“ecd,” binds EGF), transmembrane domain (“tmd,” mediates receptor dimerization), and two distinct tyrosine residues (“Y1” and “Y2,” can be phosphorylated or involved in binding). Sites shown in yellow must be unbound; white indicates sites irrelevant for the rule (tyrosines can be phosphorylated/unphosphorylated and bound/unbound, as indicated by “?”). **(B)** Four explicit reactions generated by this rule, demonstrating all combinations of tyrosine phosphorylation states. “Mem” denotes membrane localization; “ec” denotes extracellular space.

**FIGURE 3:**
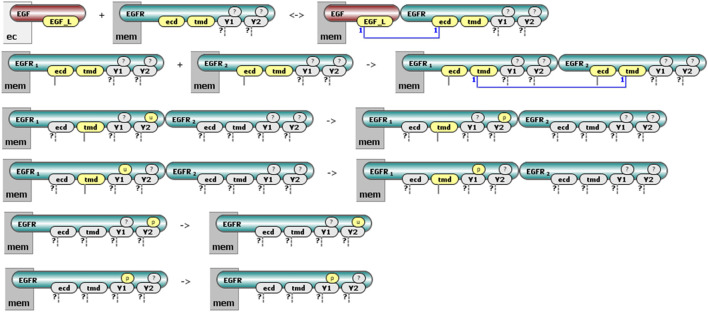
Initial steps in EGFR signaling visualized in VCell cartoons. The steps include: (1) EGF ligand binding to EGFR, conditional only on the extracellular binding site availability; (2) receptor dimerization, conditional on ligand binding to extracellular sites; (3) transphosphorylation of receptor tyrosines conditional on transmembrane dimerization; and (4) dephosphorylation of tyrosines.

Rule-based modeling overcomes limitations posed by conventional reaction network approaches by providing a compact and systematic method to represent biomolecular interactions. In this framework, a model is defined by reaction rules—precise descriptions of biomolecular transformations and interactions based on specific molecular features. Rather than explicitly defining every molecular species, each rule defines patterns of molecular states or features required for reactions, enabling a single rule to represent numerous individual reactions (Figure 2). Figure 3 illustrates the steps in EGFR signaling using rule-based modeling conventions implemented in VCell (Schaff et al., 2016).

Rule-based models can be automatically translated into conventional reaction networks by software tools such as BioNetGen (Blinov et al., 2004; Faeder et al., 2009; Harris et al., 2016). This automated generation reduces the burden of explicitly enumerating every possible molecular species, facilitating the execution of time course simulations. Simulation results can subsequently be processed to yield relevant observables, such as the total abundance of molecules with specific phosphorylation states. For cases involving extremely large or even infinite reaction networks, “network-free” agent-based simulation techniques, as implemented in tools like NFsim (Sneddon et al., 2011), provide an efficient alternative. Other rule-based modeling software includes Simmune (Meier-Schellersheim et al., 2006), Kappa (Danos et al., 2007a; Danos et al., 2007b), rxncon (Tiger et al., 2012), PySB (Lopez et al., 2013). Rule-based modeling is also implemented in general-purpose tools such as VCell (Schaff et al., 2016) and MCell (Husar et al., 2024). Of note, beyond their computational utility, rule-based models can also serve as comprehensive repositories of molecular interaction details at the site-specific level (Thomson et al., 2011; Chylek et al., 2014).

### 2.3 Visualization of rule-based models

Visualizing rule-based models poses significant challenges due to the potentially vast size and complexity of reaction networks generated by rules. For example, a detailed EGFR signaling model involving proteins such as Shc, Grb2, and Sos (Blinov et al., 2006a) includes 3,749 interactions among 356 distinct species, precluding practical visualization as a single comprehensive reaction network.

Several visualization approaches have been proposed to address these limitations. Early methods involved depicting each reaction rule individually as a cartoon illustrating its reactants and products (Meier-Schellersheim et al., 2006; Blinov et al., 2006b; Blinov et al., 2006c). This method, implemented in tools like Simmune (Cheng et al., 2014; Zhang et al., 2013), VCell (Schaff et al., 2016) (Figure 3), and recently bnglViz (Liguori-Bills & Blinov, 2024) facilitates understanding individual rules but fails to provide global insight into overall system connectivity. Consequently, the approach is often confusing for biologists accustomed to pathway-oriented representations.

**FIGURE 4:**
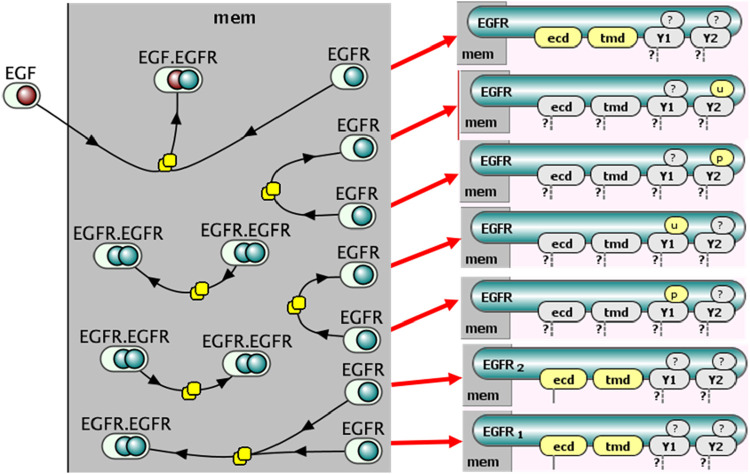
Graph representation illustrating reaction rules with distinct reactant and product patterns as separate nodes. The resulting bipartite graph is often highly disconnected due to distinct pattern definitions across rules, as illustrated by the detailed state representations of multiple monomeric EGFR states shown on the right.

Although reaction networks visually resemble pathway diagrams—since products from one reaction can become reactants in another—this is rarely the case for rule-based models. Here, reactants and products are defined by broader molecular patterns, making direct chaining between rules uncommon (Blinov and Moraru, 2012). Thus, a straightforward graphical translation of rules often results in disconnected diagrams (Figure 4).

An alternative, compact visualization is the Molecular Interaction Maps (Kohn et al., 2006) and Extended Contact Maps (Chylek et al., 2011). These maps illustrate possible interactions between molecules at the level of individual molecular sites. While effective at compactly summarizing potential molecular interactions, they fail to clearly represent the temporal or directional flow of information.

Other visualization approaches, such as rule influence diagrams (Smith et al., 2012), atom-rule graphs (Sekar et al., 2017), and the Simmune NetworkViewer (Zhang et al., 2013), aim to illustrate the relationships and influences among rules or molecular features. The NetworkViewer, for example, creates a bipartite graph linking rules to specific reactant/product patterns, closely resembling traditional pathways. However, this approach rapidly becomes impractical for models exhibiting combinatorial growth in the number of nodes when molecules have multiple binding configurations.

To address these limitations, we propose a new visualization approach based on bipartite graphs with two types of nodes: “process” nodes, representing reaction rules, and “molecular complex” nodes, representing collections of molecular species defined by shared reactant or product patterns. This mirrors the classical reaction network formalism, which also employs two node types: “reaction” nodes and “species” nodes. However, while species nodes in reaction networks represent fully defined species, “molecular complex” nodes instead encompass all possible species with a given molecular composition, as specified by the molecules included in reactant and product patterns.

We discuss this approach in more detail in the following section, but note here that, similar to classical reaction networks, the Molecular Process Diagram (MPD) formalism preserves connectivity and clarity, providing an intuitive view of information flow across modeled interactions. Importantly, it scales linearly with the number of reaction rules: the number of “process” nodes equals the number of rules, while the number of “molecular complex” nodes is bounded by the maximum number of molecular patterns in the reaction rules. This, in turn, scales with the product of the maximum number of reactants and products in a reaction rule and the total number of rules, thereby mitigating the challenge of combinatorial complexity.

## 3 Results

### 3.1 Molecular process diagrams (MPDs)

The central objective of our visualization approach is to represent rule-based models clearly and intuitively, balancing detailed rule-specific information with a coherent depiction of information flow through the system. We define a **molecular complex** as a collection of molecules specified by reactant or product patterns. For instance, in the reaction rule depicted in Figure 2, the reactant side comprises two distinct molecular complexes, while the product side is represented by a single complex.

**FIGURE 5:**
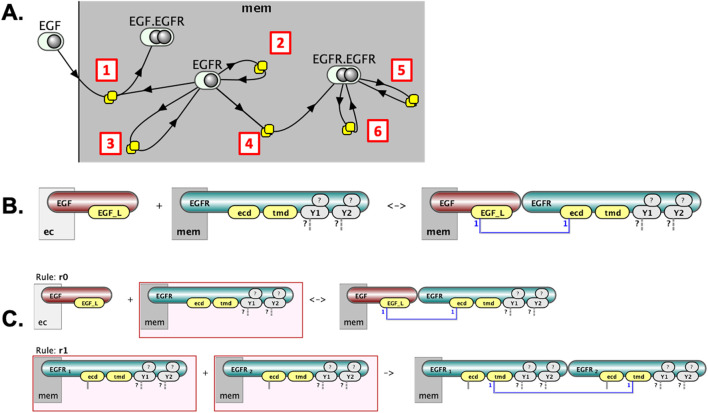
Layered visualization of a rule-based model in VCell. **(A)** The Molecular Process Diagram (MPD) provides an overview: EGF binds EGFR [1], EGFR undergoes monomeric modifications [2,3], dimerizes [4], and the EGFR dimer further undergoes modifications [5,6]. **(B)** Clicking on a process node (e.g., [1]) reveals detailed, site-specific reaction rules. **(C)** Clicking on a molecular complex node (EGFR) shows distinct state patterns in rules it participates in, highlighted by red boxes (two example rules shown).

We introduce **Molecular Process Diagrams (MPDs)** as bipartite graphs composed of two distinct node types: “molecular complexes” and “processes.” The number of process nodes equals the number of reaction rules, while the number of molecular complex nodes is typically fewer than or equal to the total number of unique reactant and product patterns across all rules. For example, the six reaction rules depicted individually in Figure 2 correspond to a compact MPD consisting of six process nodes and only four molecular complex nodes (Figure 5A).

This representation offers an intuitive, pathway-like overview. For instance, one can easily follow sequential interactions such as the binding of EGF to EGFR, the subsequent formation of receptor dimers, and the distinct modifications of EGFR molecules within dimeric or monomeric states. However, simplifying the diagram to this level involves a trade-off, as it reduces explicit representation of detailed causal relationships among molecular states.

### 3.2 Incorporating site-specific details into molecular process diagrams

To retain and display detailed, site-specific interaction information, the VCell implementation of MPDs provides interactive node expansion. Clicking on a process node reveals a detailed cartoon of the corresponding reaction rule, explicitly showing the molecular interaction details (Figure 5B). Similarly, clicking on molecular complex nodes reveals precise reactant and product patterns associated with each complex (Figure 5C). The supplemental material available on GitHub provides examples and details on building molecular process diagrams of several published models that use BioNetGen in VCell software. A bngl file can be imported into VCell and visualized immediately.

**FIGURE 6:**
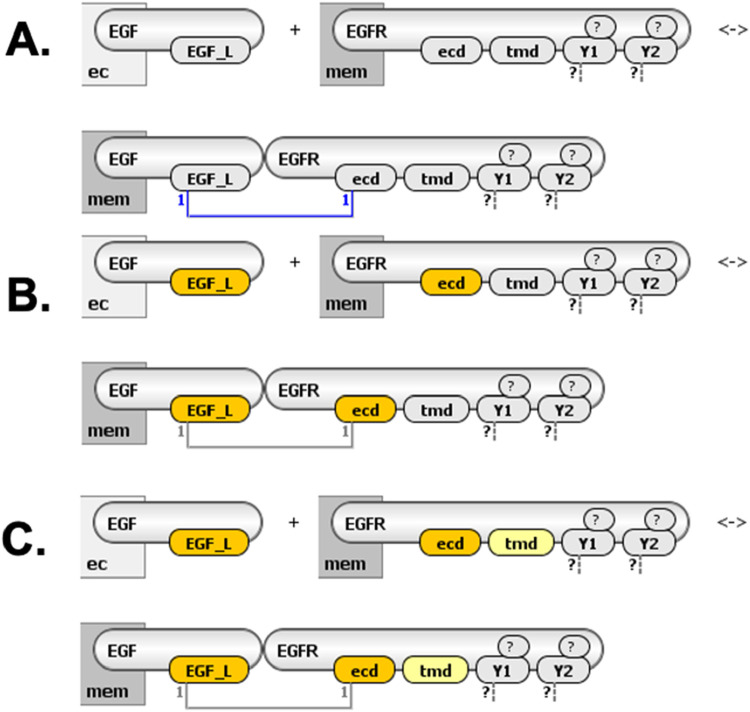
Highlighting site-specific reaction details in VCell. **(A)** Visualization without highlighting; all interacting molecules and sites displayed uniformly. **(B)** Sites undergoing modification by the reaction rule highlighted in bright yellow (e.g., receptor site “ecd” changing from “unbound” to “bound”). **(C)** Sites unchanged but required in a specific state (“molecular context”) highlighted in pale yellow (e.g., receptor site “tmd” must be unbound).

Despite these detailed visualizations, the abundance of molecular details can make identifying critical molecular features challenging. To address this, we implemented interactive highlighting options within VCell. Users can selectively highlight interacting molecules (either using distinct colors as shown in Figure 5 or uniformly gray as in Figure 6), molecular sites undergoing modification (Figure 6B), and the contextual state of molecular sites that are unchanged yet required for the interaction to occur (Figure 6C). These interactive visualization options allow users to more clearly identify and focus on key molecular features relevant to each reaction rule.

### 3.3 SBGN-compliant molecular process diagram

The Systems Biology Graphical Notation (SBGN) (Le Novère et al., 2009) provides standardized graphical conventions adopted by the modeler and developer communities for representing biological interactions and processes in a format that is both human-readable and machine-processable. Three orthogonal visual languages exist within SBGN: Process Description (PD), Entity Relationship (ER), and Activity Flow (AF). SBGN PD diagrams explicitly represent the temporal sequence of biochemical reactions; they are widely used in pathway databases (e.g., PantherDB (Mi et al., 2005), Reactome (Joshi-Tope et al., 2005), GeneXplain (Kolpakov et al., 2011)) and supported by modeling software like CellDesigner (Funahashi et al., 2003), COPASI (Hoops et al., 2006), and BIOCHAM (Calzone et al., 2006). Visualization tools compatible with the Biological Pathway Exchange (BioPAX; Demir et al., 2010) standard frequently offer SBGN-PD representations (e.g., VISIBIOweb (Dilek et al., 2010), CHiBE (Babur et al., 2014), Cytoscape plugin cySBGN (Gonçalves et al., 2013), Newt (Balci et al., 2021)). Support for SBGN visualization is facilitated by libSBGN, a dedicated software library implementing the SBGN Markup Language (SBGN-ML) (Van Iersel et al., 2012).

To represent MPDs in compliance with SBGN, we adapted existing PD diagram conventions to accommodate the site-specific information required by reaction rules. Each rule explicitly defines transformations occurring at particular molecular sites.

**FIGURE 7:**
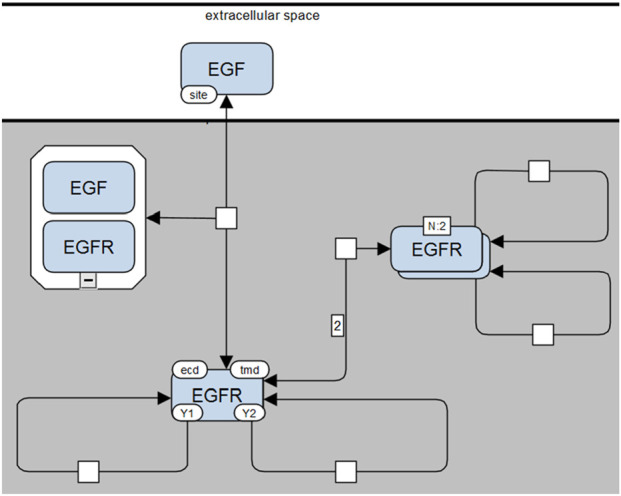
Molecular Process Diagram in SBGN-compliant notation (corresponding to the VCell representation shown in Figure 5A). EGF and EGFR represented by macromolecule glyphs; EGF-EGFR complexes use container glyphs, and EGFR dimers use multimer glyphs. Molecular sites (“site,” “ecd,” “tmd,” “Y1,” “Y2”) shown as state variable glyphs (once per molecule). Stoichiometries greater than one explicitly shown on arcs (e.g., “2” for dimerization).

**FIGURE 8:**
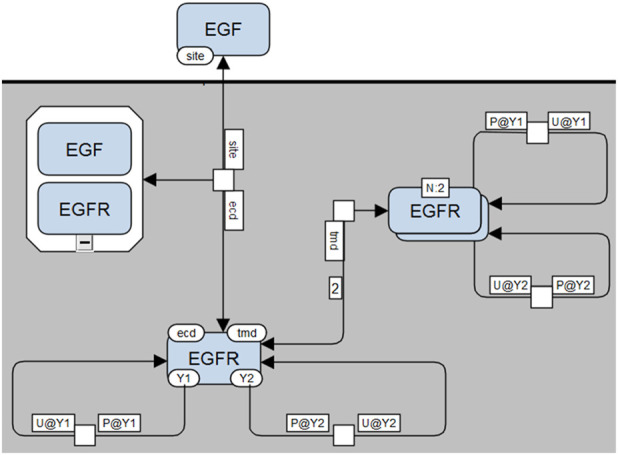
Site-specific Molecular Process Diagram visualized in SBGN notation. Shows EGF “site” binding to EGFR “ecd” site, EGFR-EGFR interactions via “tmd” sites, and phosphorylation state changes (“U” to “P” or “P” to “U”) for EGFR sites “Y1” and “Y2”, shown as in boxes along reaction arrows next to process nodes.

**FIGURE 9:**
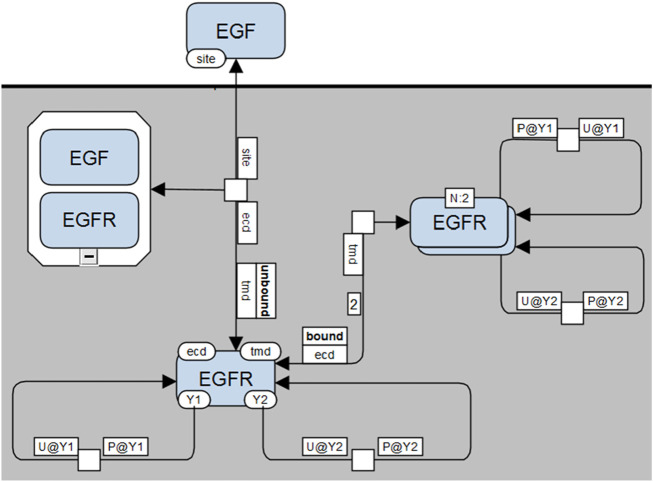
Fully detailed SBGN-compliant Molecular Process Diagram visualization. Illustrates all required molecular contexts explicitly: EGF-EGFR binding requires “tmd” sites of EGFR to be unbound, and EGFR dimerization requires “ecd” sites to be bound. These conditions are shown in extra boxes along reaction arrows.

- **Coarse-grained MPD**: The simplest MPD (Figure 7) corresponds closely to the VCell representation in Figure 5A, where complexes (e.g., receptor dimers) are depicted using SBGN multimer glyphs. Site-specific details are minimized, displayed only when explicitly involved in an interaction.
- **Intermediate MPD with site modifications**: A more detailed MPD (Figure 8) includes explicitly depicted molecular sites modified by each rule. Reactant site details are placed along consumption arcs, while product site details appear along production arcs, each labeled according to standard SBGN conventions (e.g., phosphorylation denoted as “P@Y1”). The spatial positioning of site-state information adheres strictly to SBGN guidelines, ensuring compatibility with automated processing. Binding interactions specifically display site information only on consumption arcs.
- **Detailed MPD with full contextual information**: The most detailed version (Figure 9) comprehensively depicts all site-specific information, including molecular sites required to be in particular states (bound, unbound, phosphorylated, etc.) by the process, even when unchanged by the process itself. These contextual states are presented in dedicated annotation boxes clearly labeled (e.g., “bound” or “unbound”).

This comprehensive MPD visualization allows for accurate reconstruction of the underlying rule-based model, enabling straightforward mapping from process nodes back to reaction rules, from arcs back to reactant/product patterns, and from site annotations to contextual constraints. The supplemental material available on GitHub provides examples and details on building SBGN compliant molecular process diagrams of several published models that use BioNetGen. These diagrams are implemented in yED software.

An MPD is analogous to a reaction network diagram in that it captures interactions, but it omits details needed for simulation, such as the initial species with non-zero concentrations or protocols that activate or deactivate interactions. Rule-based models go beyond a simple set of reactions, combining declarative elements (explicit molecular assumptions) with prescriptive ones (e.g., selection of seed species, kinetic laws, simulation algorithm parameters, and rule inclusion/exclusion criteria). Thus, while MPDs serve effectively as visual analogs of reaction diagrams, they do not represent the prescriptive details required for modeling.

## 4 Discussion

Dynamical models that explicitly account for the intricate details of multimolecular complexes within cells are becoming increasingly common, driven by the expanding availability of detailed biological data. Historically, cartoon diagrams—whether informal or standardized—have been essential for visualizing biological knowledge and hypotheses. Such diagrams have served as a primary means of representing biological pathways, from qualitative descriptions to quantitative mechanistic models. However, the visualization of models that consider detailed molecular interactions, particularly rule-based models, poses significant challenges. These models have become more widespread as they are inherently able to address the combinatorial complexity that arises from explicitly considering numerous possible molecular interactions, binding states, and site-specific modifications.

We developed a novel visualization approach using multilayered Molecular Process Diagrams (MPDs). MPDs offer a unified representation of rule-based models by combining a clear overview of molecular interactions (“big picture”) with explicit and precise site-specific details and conditions (“detailed assumptions”). The approach’s scalability derives from three distinct but complementary information layers: interacting molecular complexes, molecular sites modified by reactions, and molecular context—sites that, while not modified, are necessary for interactions to occur. This layered structure enables seamless navigation back and forth between general, pathway-level insights and detailed, site-specific molecular interactions, ensuring that modeling assumptions remain transparent and clearly communicated.

While in the manuscript we illustrate a relatively simple model of initial events in EGFR signaling, the MPD is able to illustrate very large rule-based models, such included in the supplemental material the model by Hat et al. (2016) that describes the complex circuitry of the p53 network regulated by Mdm2, Wip1, Wip1 and PTEN. The model has 58 rules specifying interactions among 31 molecules. The models of EGFR are well understood and this is the reason we have them as an example, but they can go in complexity up to 99 rules specifying interactions among EGF ligand, EGF receptor and 34 adapter proteins (the paper by Stites et al., 2015), and the model in the paper by Creamer et al., 2012 describes signaling events in ERBB signaling leading to activation of ERK and Akt. It tracks phosphorylation of 55 individual serine, threonine, and tyrosine residues, and includes 544 rules specifying interactions among 19 molecules. Even such a huge model can be visualized effectively using the different graph layout algorithms that we developed for VCell, providing a connectivity overview that can highlight the importance of specific molecular complexes. Naturally, visualizing a model with 544 rules as a single cartoon is of limited value. However, opening the model in the software and exploring each connected component by zooming in can provide a useful bird’s-eye view of the system.

An important consideration is that the content and organization of MPDs depend explicitly on the formulation of the underlying reaction rules. Specifically, molecules displayed within complex nodes reflect exactly how these complexes are described by the reactant and product patterns in the rules, rather than being derived from broader network-level analyses. Consequently, alternative formulations of rules, such as the use of bond wildcards or explicit specification of bound molecules, can significantly alter the resulting MPD, while not changing the model. For example, reaction rules can be made very concise, a ligand-receptor complex can be shown just as a receptor in a bound state. However, for visualization purposes it may be more useful to alter the model to include ligand explicitly in all rules that include ligand-receptor complexes. While mathematically the model remains the same, visualization may become more intuitive.

The MPD approach has been fully implemented within the widely used VCell modeling and simulation framework. The associated graphical user interface (GUI) provides biologists with a user-friendly, intuitive method for constructing and analyzing rule-based models, clearly specifying the details and spatial localization of each interaction rule.

We have further demonstrated how the existing Systems Biology Graphical Notation (SBGN) standards can be adapted with minimal alterations to effectively visualize MPDs. Importantly, the SBGN-compliant MPDs introduced here (Figures 7–9) maintain compatibility with standard SBGN Process Description (PD) diagrams, as removal of additional site-specific annotations results in a conventional SBGN-PD representation. Given that the syntax and semantics of SBGN are community-driven, our proposed extensions will require ongoing discussion and validation within the SBGN research community. Nonetheless, we anticipate that our approach provides a straightforward and easily adoptable solution for integrating detailed rule-based information within the existing standard. For broader adoption, rule-based elements should be incorporated into the SBGN standard, and tools using SBGN must be able to support these new notations. Achieving the first step requires a community-wide discussion, since SBGN is a standard intended for adoption by both the systems biology community and those interested in visualization. While adapting backend tools such as libSBGN (Van Iersel et al., 2012) is usually straightforward, developing intuitive user interfaces and achieving proper rendering is considerably more challenging. One possible solution is to employ GraphML, which is supported by various visualization tools. We are currently working on designing the most appropriate schema for its implementation.

Finally, while the MPD concept was originally designed to address the specific complexities associated with visualizing rule-based models, its utility is not limited to this context. MPDs offer a valuable visualization tool for any scenario requiring detailed graphical representation of site-specific interactions among biomolecules, potentially extending its applicability across diverse areas of systems biology and molecular modeling.

## Data Availability

The datasets presented in this study can be found in online repositories. The names of the repository/repositories and accession number(s) can be found below: https://github.com/vcellmike/MolecularProcessDiagram.
